# Evolutionary Consequences of DNA Methylation on the GC Content in Vertebrate Genomes

**DOI:** 10.1534/g3.114.015545

**Published:** 2015-01-15

**Authors:** Carina F. Mugal, Peter F. Arndt, Lena Holm, Hans Ellegren

**Affiliations:** * Department of Ecology and Genetics, Uppsala University, SE-752 36 Uppsala, Sweden; † Department of Computational Molecular Biology, Max Planck Institute for Molecular Genetics, DE-14195 Berlin, Germany; ‡ Department of Anatomy, Physiology and Biochemistry, Swedish University of Agricultural Sciences, SE-756 51 Uppsala, Sweden

**Keywords:** DNA methylation, CpG hypermutability, GC isochores, GC content, GC-biased gene conversion

## Abstract

The genomes of many vertebrates show a characteristic variation in GC content. To explain its origin and evolution, mainly three mechanisms have been proposed: selection for GC content, mutation bias, and GC-biased gene conversion. At present, the mechanism of GC-biased gene conversion, *i.e.*, short-scale, unidirectional exchanges between homologous chromosomes in the neighborhood of recombination-initiating double-strand breaks in favor for GC nucleotides, is the most widely accepted hypothesis. We here suggest that DNA methylation also plays an important role in the evolution of GC content in vertebrate genomes. To test this hypothesis, we investigated one mammalian (human) and one avian (chicken) genome. We used bisulfite sequencing to generate a whole-genome methylation map of chicken sperm and made use of a publicly available whole-genome methylation map of human sperm. Inclusion of these methylation maps into a model of GC content evolution provided significant support for the impact of DNA methylation on the local equilibrium GC content. Moreover, two different estimates of equilibrium GC content, one that neglects and one that incorporates the impact of DNA methylation and the concomitant CpG hypermutability, give estimates that differ by approximately 15% in both genomes, arguing for a strong impact of DNA methylation on the evolution of GC content. Thus, our results put forward that previous estimates of equilibrium GC content, which neglect the hypermutability of CpG dinucleotides, need to be reevaluated.

DNA methylation is a common feature of vertebrate genomes and predominantly occurs at cytosines in CpG dinucleotides and converts cytosine into 5-methylcytosine (Bird and Taggart 1980); it also may occur at different sequence contexts although at a much lower frequency (Lister *et al.* 2009). Its function mainly has been associated with transcriptional regulation (Jones 2012). Beside its prominent role in transcriptional regulation, the DNA methylation pattern that is present in the germline has the potential to leave an evolutionary signature in the genome, as the mutability of methylated cytosines is approximately one order of magnitude greater than that of nonmethylated cytosines (Holliday and Grigg 1993). This increased mutability is often referred to as CpG hypermutability, and its evolutionary consequence is a depletion of CpG dinucleotides (Bird 1980; Cooper and Krawczak 1989), thereby influencing regional base composition and the evolution of GC content.

Mainly, three mechanisms have been discussed in relation to the evolution of GC content and its characteristic variation across the genome (Eyre-Walker and Hurst 2001). When the variation in GC content across the genome was first discovered, it was thought that the genome was arranged in discrete segments of homogeneous GC content that were separated by borders of sharp transition, the so-called GC isochore structure (Filipski *et al.* 1973). As GC-rich isochores were found to be more abundant in warm-blooded than in cold-blooded vertebrates, it was proposed that natural selection could act upon the thermal stability of DNA (Bernardi *et al.* 1985; Bernardi 1990, 2007); GC-rich DNA is more thermally stable than AT-rich DNA. Alternatively, natural selection for variation in GC content could assist the regulation of gene expression, because the local GC content is associated with chromatin structure and the distribution of genes (Aissani and Bernardi 1991; Duret *et al.* 1995).

However, the importance of natural selection in the context of evolution of base composition has been questioned, and variation in GC content has been shown to be less drastic, with continuous transitions between large regions of the genome that show local similarities in GC content (Li 2002). Two neutral mechanisms have subsequently been put forward to explain the variation in GC content across the genome: mutation bias and GC-biased gene conversion (gBGC). The mutation bias hypothesis posits that variation in GC content arises through variation in mutation patterns across the genome. For example, mutation patterns could vary with replication timing as free nucleotide concentrations, which vary during S phase, show an impact on base misincorporation during DNA replication (Wolfe *et al.* 1989; Kumar *et al.* 2011).

An alternative explanation for a mutation bias was built on the observation that the cytosine deamination rate and GC content affect each other (Fryxell and Zuckerkandl 2000). At the same time as spontaneous cytosine deamination provokes most C → T substitutions in vertebrate genomes and acts to reduce the GC content, high GC content increases the stability of double-stranded DNA and acts rate-limiting for cytosine deamination. Thus, cytosine deamination rate should be lower in GC-rich than in GC-poor regions, potentially leading to a positive feedback loop between cytosine deamination rate and GC content that could allow for a reinforcement of the heterogeneity in GC content across the genome.

The gBGC hypothesis suggests that variation in GC content arises through variation in recombination rate across the genome. gBGC is a mechanism related to meiotic recombination, which leads to a preferential fixation of GC-alleles over AT-alleles at AT/GC heterozygous sites close to recombination-initiating double-strand breaks (Duret and Galtier 2009). As a consequence, high-recombining regions tend to show a greater GC content than low-recombining regions. Several lines of evidence suggest that gBGC is a key player in the evolution of GC content (Webster *et al.* 2006; Duret and Arndt 2008; Romiguier *et al.* 2010), and the gBGC hypothesis is currently favored by many authors. However, the different hypotheses are not necessarily mutually exclusive, and two or all three of them might act together on the evolution of the GC content, as well as other factors might be involved.

Here we focus on the potential impact of DNA methylation and the concomitant CpG hypermutability on the evolution of GC content in vertebrate genomes. For this purpose, we generated a whole-genome methylation map of chicken sperm cells by bisulfite sequencing and made use of a publicly available whole-genome methylation map of human sperm cells (Molaro *et al.* 2011). We incorporated data from these maps with estimates on recombination rate into a model of GC content evolution in order to investigate the impact of DNA methylation on the local equilibrium GC content (GC*).

## Material and Methods

### Sequence data

Whole-genome sequence alignments for chicken, turkey, and zebra finch and for human, macaque, and mouse were retrieved from the Ensembl database release 73 via the Ensembl perl Application Program Interfaces. Alignments were based on the EPO pipeline, where avian alignments included only the three named species whereas mammalian alignments were based on the 13 Eutherian mammals. We partitioned the whole-genome alignments into consecutive, nonoverlapping windows of 1 Mb, where for the respective group of three species partitioning was performed with reference to the chicken or human genome. Positions of transcribed regions including untranslated regions and repetitive sequences were established and masked from the alignments. Transcribed regions and untranslated regions coordinates were obtained through the BioMart query interface (http://www.ensembl.org/biomart/martview). Annotation of repetitive sequences was based on the RepeatMasker program and positions of repetitive sequences were retrieved from the Ensembl database release 73. Finally, we restricted the data to windows with a minimum of 10,000 unambiguous sites, of which there were 1005 in the chicken reference and 1655 in the human reference.

### Estimation of nucleotide substitution rates

We estimated lineage-specific nucleotide substitution rates for intergenic regions using a maximum likelihood approach (Duret and Arndt 2008). In this framework triple alignments of two sister species (in our case, chicken and turkey or human and macaque) with one outgroup species (zebra finch or mouse) are taken and a general model of sequence evolution is fitted to these data. This probabilistic model does not assume stationarity of the nucleotide substitution process, accounts for multiple hits, distinguishes six reverse complement symmetric nucleotide exchanges, incorporates neighbor dependency due to the prevalent methyl-cytosine deamination process at CpG dinucleotides (CpG → CpA/TpG), and is lineage-specific. In other words, it models the two branches to the sister species independently.

On the basis of this model we then computed W → S, S → W, S → S, and W → W nucleotide substitution rates for chicken and human, respectively, where W indicates “weak” nucleotides (A, T) and S “strong” nucleotides (C, G). To summarize, X → Y substitution rate represents the number of changes along a specific branch from nucleotides X to nucleotides Y per nucleotide of type X. For example, chicken-specific W → S nucleotide substitution rate gives the number of changes along the chicken branch, subsequent to the split from turkey, from A or T to G or C per “weak” nucleotide site. Furthermore, to avoid that nucleotide substitution rate variation and specifically S → W nucleotide substitution rate variation are caused by hypermutability of CpG dinucleotides and thus affected by the local CpG content and DNA methylation level, changes of the type CpG → CpA/TpG are considered separately.

### Estimation of GC^*^ and GC^*^_CpG_

The estimation of lineage-specific substitution rates allowed us to compute the GC content at equilibrium. We defined two quantities of equilibrium GC content, GC* and GC*_CpG_. GC* reflects the equilibrium GC content without CpG hypermutability. As such, GC* is given by the following:

$$GC^{*}=\frac{u_{W\to S}}{u_{W\to S}+u_{S\to W}},$$

Here *u_W → S_* represents the W → S substitution rate, and *u_S → W_* represents the S → W substitution rate, where for the latter changes of the type CpG → CpA/TpG were discarded. GC*_CpG_ reflects the equilibrium GC content, which takes CpG hypermutability into account and was computed by a cluster approximation method as described previously (Arndt *et al.* 2003).

### Whole-genome methylation maps

DNA was extracted from 100 µL of chicken sperm, directly frozen upon sampling. Thawed sperm cells were washed twice in water and 200 µL of Laird’s lysis buffer (Tris-Col 100mM, ethylenediaminetetraacetic acid 5 mM, NaCal 20 mM, sodium dodecyl sulfate 2%, dithiothreitol 40 mM, and proteinase K 500 µg/mL) was then added and was incubated overnight at 50°. The sample was then purified by two rounds of phenol-chloroform/chloroform extraction followed by ethanol precipitation. Fragmentation of the purified DNA to a mean size of approximately 200 bp (range 100−300 bp) was done by sonication and was followed by end repair, dA addition to 3′-end and ligation of methylated sequencing adaptors following the Illumina Paired-End protocol. Ligated DNA was bisulfite converted, *i.e.*, conversion of nonmethylated cytosines to uracil, using the EZ DNA Methylation-Gold Kit (Zymo Research) according to the manufacturer’s instructions, which includes final desulfonation. The resulting fragments were subsequently size selected to yield two final libraries of mean insert size of 334 bp and 375 bp, respectively, and amplified before cluster preparation and sequencing on a Illumina HiSeq instrument, following the manufacturer’s protocol. Read length was 90 bp.

Sequences from the two libraries were pooled and filtered by removing adaptor sequences, contamination, and low-quality reads from raw reads. Filtered reads were then aligned to the WASHUC2 assembly version of the chicken genome using SOAPaligner-v2.21 (Luo *et al.* 2012). The number of reads covering each CpG site were separated into those corresponding to ^m^C (*i.e.*, nonconverted upon bisulfite treatment) and those corresponding to C (*i.e.*, converted upon bisulfite treatment).

Human processed data files were downloaded from the NGSmethDB database (http://bioinfo2.ugr.es/NGSmethDB/database.php), sample spermdonor1 and #reads>=1 (Molaro *et al.* 2011). For each CpG site, the number of reads covering that CpG site corresponding to ^m^C (*i.e.*, nonconverted upon bisulfite treatment) and the total number of reads covering that CpG site were listed.

Methylation status of each CpG site was specified as the number of reads corresponding to ^m^C (denoted as *x_mCpG_*) divided by the total number of reads covering each cytosine site in the reference (denoted as *x_mCpG_* + *x_CpG_*). Methylation status thus ranged between 0 and 1. CpG methylation level per window was then determined as the average methylation status of CpGs,

$$L_{\mathrm{mCpG}}=\frac{\sum_{\mathrm{CpG}\in \Omega }x_{\mathrm{mCpG}}}{\sum_{\mathrm{CpG}\in \Omega }\left(x_{\mathrm{mCpG}}+x_{\mathrm{CpG}}\right)}.$$

Here, Ω represents the set of all CpG dinucleotides in nontranscribed and nonrepetitive regions within each window.

### Estimation of recombination rate

We estimated sex-specific and sex-averaged chicken recombination rate for each 1-Mb window using data from Groenen *et al.* (2009). Recombination rate was approximated by the mean crossover rate between pairs of markers weighted by the physical distance between them. Sex-specific and sex-averaged human recombination rate estimates were retrieved from the University of California, Santa Cruz genome browser based on the most recent deCode genetic map (Kong *et al.* 2010), where again recombination rate was approximated by crossover rate.

### Regression analysis

All regression analyses were based on 857 of 1005 windows in the chicken genome and 1048 of 1655 windows in the human genome, where data on all considered candidate explanatory variables and response variables were available. We performed multiple linear regression (MLR) analysis using CpG methylation level and recombination rate as candidate explanatory variables for CpG → CpA/TpG substitution rate. We transformed recombination rate estimates to reduce the skewness in their distribution by log-transformation to base 10, after adding a constant of 1. We further performed MLR analysis using the frequency of methylated sites, CpG[o/e], and recombination rate as candidate explanatory variables for GC*_CpG_ and ΔGC = GC*_CpG_ – GC, respectively. Recombination rate estimates were transformed as described previously. Because the explanatory variables were highly correlated with each other and MLR analysis is sensitive to multicollinearity among explanatory variables, we also performed principal component regression (PCR) analysis after further Z-transformation of the explanatory variables, which means standardization of the mean value to 0 and of the standard deviation to 1. First, PCR groups together explanatory variables into principal components (PCs) based on their correlations with each other, whereas subsequent regression analysis and the number of significant PCs illustrates the number of independent effects on the response variable. Each significant principal component (PC) represents an independent effect by at least one of the contributors to the respective PC on the response variable. All regression analyses were performed with the software package R version 2.7.2.

## Results

### DNA methylation and CpG hypermutability

We generated a genome-wide methylation map for chicken sperm cells at single base-pair resolution by bisulfite sequencing. From a total of 419.8 million raw reads (37.8 Gb of sequence), 324.4 million reads (29.2 Gb) could be mapped to the chicken genome, corresponding to a mean coverage of 28X. On the basis of these data, we retrieved the methylation status for all covered CpG dinucleotides. CpG methylation level was then determined as the average CpG methylation status in 1-Mb windows based on nontranscribed and nonrepetitive regions of the genome. We further determined CpG methylation level for human sperm cells based on previously published data (Molaro *et al.* 2011). We then computed GC content, CpG content, and CpG[o/e], *i.e.*, the ratio of observed *vs.* expected CpG content, for chicken and human. Genome-wide averages and minimum and maximum values of these four genomic features are listed in Table 1. We found an overall lower CpG methylation level in chicken (41%) than in human (70%), whereas the values of GC content, CpG content, and CpG[o/e] were similar between the bird and mammalian representatives.

**Table 1:** Genome-wide averages (range) of CpG methylation level, GC content, CpG content and CpG[o/e]

	Chicken	Human
CpG methylation level	0.41 (0.18–0.53)	0.70 (0.22–0.92)
GC content	0.4034 (0.3224–0.5509)	0.4090 (0.2939–0.6444)
CpG content	0.0092 (0.0032–0.0316)	0.0093 (0.0029–0.0445)
CpG[o/e]	0.21 (0.12–0.42)	0.20 (0.10–0.50)

Genome-wide averages (range) were determined based on nontranscribed and nonrepetitive regions of the genome.

To explore the impact of DNA methylation on the hypermutability of CpG dinucleotides, we estimated CpG → CpA/TpG substitution rate and analyzed its relationship with CpG methylation level. Note that CpG methylation level was derived from sperm cells and thus represents male germ-line methylation level. Because of the lack of data on methylation levels from oogenesis, female germline methylation level was not included as an explanatory variable. Since there might be a significant contribution of maternally originating CpG mutations to the CpG substitution rate, the observed relationship between CpG → CpA/TpG substitution rate and male germline methylation level might therefore only partly describe the true relationship. We further considered recombination and incorporated sex-averaged and separately female and male recombination rates as candidate explanatory variables into a linear regression model to test the impact of gBGC on the CpG → CpA/TpG substitution rate. However, because gBGC is assumed to occur in both female and male germline and because differences in the impact of female and male recombination rates on CpG → CpA/TpG substitution rate were of minor importance (Supporting Information, Table S1 and Table S2), in the following only sex-averaged recombination rate was considered. The MLR analysis showed a positive relationship between CpG → CpA/TpG substitution rate and CpG methylation level and a negative relationship between CpG → CpA/TpG substitution rate and recombination rate in both species, Table 2. The positive relationship between CpG → CpA/TpG substitution rate and CpG methylation level was stronger in human than in chicken, which could be related to a wider range in CpG methylation level in human (0.22–0.92) compared to chicken (0.18–0.53). The negative relationship between CpG → CpA/TpG substitution rate and recombination rate was on the other hand stronger in chicken than in human. Here, the difference seems too large to be solely explained by a higher variance in recombination rate in chicken (0–3.4) compared to human (0–2.1). An alternative explanation could be a more stable recombination landscape in birds compared to mammals (Ellegren 2013), which has been suggested to reinforce a steady build-up of correlations with recombination rate (Mugal *et al.* 2013).

**Table 2:** MLR analysis of CpG → CpA/TpG substitution rate in relation to CpG methylation level and sex-averaged recombination rate

	Chicken		Human	
	Partial Correlation	*P*-Value	Partial Correlation	*P*-Value
CpG methylation level	**0.272**	5.09·10^−16^	**0.370**	< 2·10^−16^
Recombination rate	**−0.535**	< 2·10^−16^	−0.055	7.61·10^−2^
	R^2^ = 0.36		R^2^ = 0.14	

Partial correlations significant below a *P*-value threshold of 0.05 are in bold. MLR, multiple linear regression.

### The evolution of GC content

The main purpose of our study was to investigate the impact of DNA methylation, via hypermutability of CpG dinucleotides, on the evolution of the GC content. To address this question, we obtained two different estimates of local equilibrium GC content, one that neglects the hypermutability of CpG dinucleotides but instead removes CpG dinucleotides from the genome and one that incorporates the hypermutability of CpG dinucleotides. The former equilibrium GC content is referred to as GC* and the latter as GC*_CpG_. Genome-wide averages of these two estimates (Table 3) showed that incorporation of CpG hypermutability reduces estimates of equilibrium GC content of approximately 16% in chicken and 14% in human. We find that CpG hypermutability in chicken acts to keep the GC content at its current mean, whereas GC content would be increasing under a model of GC content evolution that neglects CpG hypermutability. In human, CpG hypermutability leads to a decrease in GC content over time, while GC content would be kept at its current mean otherwise.

**Table 3:** Genome-wide averages (range) of current GC content, GC*, and GC*CpG

	Chicken	Human
GC content	0.4034 (0.3224–0.5509)	0.4090 (0.2939–0.6444)
GC*	0.4752 (0.3277–0.7440)	0.4027 (0.2056–0.7016)
GC*_CpG_	0.3988 (0.2895–0.6056)	0.3464 (0.1866–0.5725)

Genome-wide averages (range) were determined based on nontranscribed and nonrepetitive regions of the genome.

**Figure 1:**
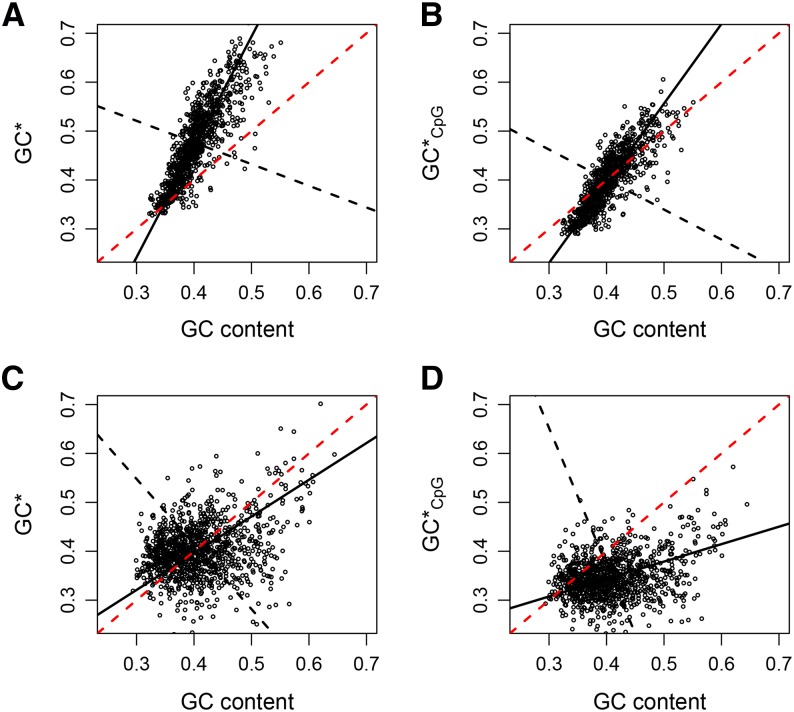
Pair-wise relationships between GC* and current GC content as well as GC*_CpG_ and current GC content (panels A and B for chicken and panels C and D for human). The black solid line represents the leading principal component fitted to the data. The intersection between the black solid and black dashed line indicates the mean values of GC* and GC content, respectively. The red dashed line represents the bisecting line of the first quadrant (x = y).

The evolution of GC content in the chicken and human genomes is further illustrated in Figure 1. In this figure, GC* and GC*_CpG_ are plotted as functions of the current GC content and the leading principal component, *i.e.*, the direction of the maximum common variation, is fitted to the data (black solid line). A slope > 1 (as found in chicken) indicates a reinforcement of the variation in GC content across the genome, whereas a slope < 1 (as found in human) indicates an erosion of the variation in GC content across the genome. Interestingly, a comparison of the slopes between the two different estimates of equilibrium GC content indicates that the reinforcement and erosion of the variation in GC content, respectively, are not affected by CpG hypermutability. In other words, DNA methylation seems to act rather uniformly across the genome to reduce the GC content.

To investigate the impact of DNA methylation in more detail, we incorporated DNA methylation into a model of GC content evolution. The impact of DNA methylation on the evolution of GC content in a genomic region should depend mainly on the frequency of methylated sites in a region, more so than on the CpG methylation level itself. We therefore computed the frequency of methylated sites per window as the product of CpG methylation level and CpG content. Genome-wide averages of methylation frequency were 0.0037 (range: 0.0008–0.0103) in chicken and 0.0062 (0.0011–0.0305) in human. In addition to methylation frequency, CpG[o/e] might provide information on the frequency of possible target sites for CpG hypermutability and was therefore also considered in the model. Thus, to investigate the importance of DNA methylation and CpG hypermutability as compared with gBGC we performed linear regression analysis of GC*_CpG_ by using methylation frequency, CpG[o/e], and recombination rate as candidate explanatory variables (Table 4).

**Table 4:** MLR analysis of GC*_CpG_ and ΔGC in relation to methylation frequency, CpG[o/e], and recombination rate

	GC*_CpG_				ΔGC			
	Chicken		Human		Chicken		Human	
	Partial Correlation	*P*-Value	Partial Correlation	*P*-Value	Partial Correlation	*P*-Value	Partial Correlation	*P*-Value
Methylation frequency	**0.174**	2.99·10^−7^	**0.208**	1.20·10^−11^	**-0.375**	< 2·10^−16^	**-0.371**	< 2·10^−16^
CpG[o/e]	**0.580**	< 2·10^−16^	0.020	5.11·10^−1^	**0.571**	< 2·10^−16^	0.001	9.79·10^−1^
Recombination rate	**0.254**	4.59·10^−14^	**0.149**	1.20·10^−6^	**0.142**	3.06·10^−5^	**0.081**	8.97·10^−16^
	R^2^ = 0.84		R^2^ = 0.17		R^2^ = 0.48		R^2^ = 0.29	

Partial correlations significant below a *P*-value threshold of 0.05 are in bold. MLR, multiple linear regression.

As predicted by gBGC we found a positive relationship between GC*_CpG_ and recombination rate. Note, however, that gBGC does not predict a linear relationship between GC*_CpG_ and the logarithm of recombination rate, as assumed by the underlying model of the linear regression analysis. Thus, the positive relationship between GC*_CpG_ and recombination rate might in fact be underestimated. CpG[o/e] showed a strong positive relationship in chicken but was of minor importance in human. Note that CpG[o/e] and GC content are positively correlated with each other due to a mathematical artifact (Pearson correlation coefficient ρ = 0.83 and ρ = 0.70 for chicken and human, respectively) (Duret and Galtier 2000). Given the absence of a strong impact of CpG[o/e] on GC*_CpG_ in human and the much stronger correlation between equilibrium and current GC content in chicken than in human, the strong positive relationship between CpG[o/e] and GC*_CpG_ found in chicken is likely caused by the transitive nature of correlations and of no biological relevance. The observed positive relationship between GC*_CpG_ and methylation frequency seems to argue against a reduction of GC content due to DNA methylation. However, regions high in GC content also provide more targets for DNA methylation, which leads to a positive correlation between current GC content and methylation frequency, and because current and equilibrium GC content are correlated with each other consequently also between GC*_CpG_ and methylation frequency. Thus, the difference between equilibrium and current GC content ΔGC = GC*_CpG_ – GC should be a more appropriate measure to explore the impact of DNA methylation on GC content evolution. We therefore repeated the linear regression analysis with ΔGC as response variable and found a negative relationship between ΔGC and methylation frequency (Table 4). The relationship between GC*_CpG_ and recombination rate remained positive. The impact of CpG[o/e] was again of minor importance in human but showed a strong positive relationship in chicken.

**Figure 2:**
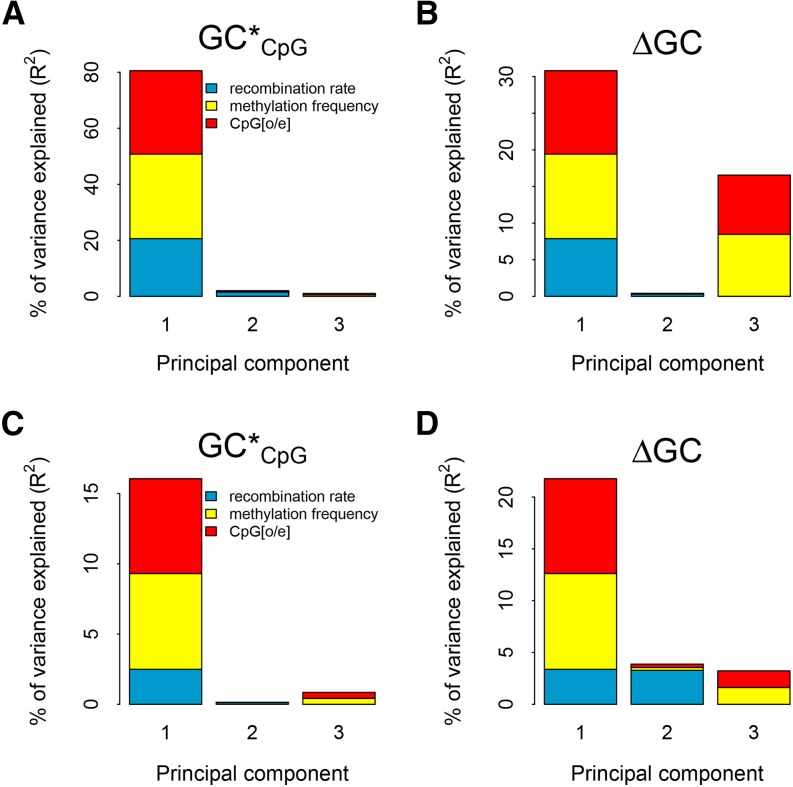
Amount of variation in GC*_CpG_ as well as ΔGC explained by the different explanatory variables based on principal component regression analysis (panels A and B for chicken and panels C and D for human, respectively). The height of each bar represents how much of the variance in GC* or ΔGC, respectively, is explained by the corresponding principal component. The size of each colored area is proportional to the relative contribution of the respective genomic feature within each principal component.

Because all three explanatory variables were strongly correlated with each other (Table 5), the inference of the relative impact of the explanatory variables needs caution. To obtain a clearer picture we therefore performed PCR analysis, which confirms that the impact of DNA methylation and recombination rate on GC content evolution is tightly linked (Figure 2). Only one principal component, PC I, which consists of all three explanatory variables, shows an impact on GC*_CpG_. For ΔGC, PCR allows us to disentangle two independent effects, PC I and PC III, in chicken and three independent effects in human, PC I, PC II, and PC III. Methylation frequency and CpG[o/e] cluster together in PC III, which shows a significant negative relationship between ΔGC and methylation frequency, and a significant positive relationship between ΔGC and CpG[o/e]. In conclusion, the PCR provides support for a reduction in GC content due to DNA methylation and CpG hypermutability.

**Table 5:** Pearson correlation coefficients between methylation frequency, CpG[o/e], and recombination rate for chicken (lower left) and human (upper right)

	Methylation Frequency	CpG[o/e]	Recombination Rate
Methylation frequency	−	0.79	0.30
CpG[o/e]	0.91	−	0.29
Recombination rate	0.58	0.56	−

All *P*-values < 2e-16.

## Discussion

### DNA methylation and the evolution of GC content

Vertebrate genomes are depleted in CpG dinucleotides as a consequence of an increased mutation rate of methylated CpG dinucleotides (Bird 1980). Consistent with this finding, we found a positive relationship between CpG → CpA/TpG substitution rate and CpG methylation level. Moreover, as estimates of germline CpG methylation level were based on sperm cells and provided that methylation patterns may very well differ between male and female germline, the observed relationship between CpG → CpA/TpG substitution rate and CpG methylation level might in fact be underestimated. The observation of a CpG[o/e] of approximately 0.20 indicates a depletion of CpG dinucleotides of about 80% in both chicken and human. Furthermore, our analysis suggested that DNA methylation and the concomitant CpG hypermutability lead to a reduction in equilibrium GC content of approximately 15% in both genomes. Thus, both gBGC and DNA methylation seem to play an important role in the evolution of GC content. On the other hand, the degree of heterogeneity in GC content across the genome seems not affected by DNA methylation. This finding appears surprising, given that GC-rich regions provide more targets for methylation and show a greater frequency of methylated sites than GC-poor regions (Caccio *et al.* 1997). A positive relationship between CpG → CpA/TpG substitution rate and CpG methylation level together with a greater frequency of methylated sites in GC-rich regions than GC-poor regions should act to homogenize the GC content across the genome, leading to an erosion of the variation in GC content. However, at the same time as DNA methylation increases the mutability of CpG dinucleotides, GC content acts to stabilize it (Fryxell and Moon 2005; Mugal and Ellegren 2011). Because GC content and DNA methylation show a positive relationship with each other, we propose that the effects of DNA methylation and GC content on cytosine mutability balance each other, providing a possible explanation to why CpG hypermutability acts rather uniformly across the genome to reduce the GC content.

The finding of a relatively uniform effect of CpG hypermutability across the genome questions the hypothesis that a positive feedback loop between cytosine deamination rate and GC content allows for a reinforcement of the variation in GC content (Fryxell and Zuckerkandl 2000). Because of the lack of DNA methylation data, this hypothesis was built solely on the effect of GC content on cytosine mutability. The availability of whole-genome DNA methylation data now demonstrates that DNA methylation acts in the opposite direction due to its positive relationship with GC content. Hence, altogether CpG hypermutability does not affect the degree of heterogeneity in GC content across the genome. As previously suggested, the degree of heterogeneity in GC content seems to be explained by the evolutionary stability in the recombination landscape (Auton *et al.* 2012; Mugal *et al.* 2013). The reinforcement of the variation in GC content in chicken and its erosion in human are thus in good agreement with a more stable recombination landscape in birds compared to mammals (Ellegren 2013), and seem not affected by GC content and DNA methylation.

### Estimation of equilibrium GC content

For nearly 20 years, nonhomogeneous, nonstationary Markov models of nucleotide substitutions have been developed and implemented to estimate lineage-specific substitution rates for an underlying phylogenetic tree (Galtier and Gouy 1998; Arndt 2007; Dutheil and Boussau 2008). Such estimates enable the study of base composition evolution by providing estimates of base composition not only for individual lineages but also for ancestral nodes and at equilibrium. The interest in the origin and evolution of the variation in GC content across the genome has given rise to a series of studies of the evolution of GC content for various groups of species [*cf*. (Belle *et al.* 2004; Duret and Arndt 2008; Romiguier *et al.* 2010; Nabholz *et al.* 2011; Lartillot 2013; Mugal *et al.* 2013)]. In most of these studies, nucleotide sites have either been assumed to evolve independent from each other or CpG dinucleotides have been excluded from the analysis [but see (Duret and Arndt 2008)]. However, dependent on the underlying nucleotide substitution model, different estimates of GC content may be obtained for the underlying phylogenetic tree. Because CpG hypermutability is prevalent in vertebrate genomes, our results emphasize the importance of using neighbor-dependent models that include CpG hypermutability when analyzing GC content evolution and estimating equilibrium GC content.

We find that in chicken the GC content seems to have reached its equilibrium and is kept at its current mean if CpG hypermutability is incorporated into the nucleotide substitution model. In contrast, GC content would be increasing under a model that neglects CpG hypermutability, as reported previously Mugal *et al.* (2013). In human, CpG hypermutability leads to a decrease in GC content over time, whereas GC content would be kept at its current mean under a model of GC content evolution that neglects CpG hypermutability. Our analysis thus suggests that previous studies on the dynamics of GC content, which have not properly accounted for CpG hypermutability, might need to be reevaluated.

## Supplementary Material

Supporting Information
