# Supplementary material for: Evolutionary Consequences of DNA Methylation on the GC Content in Vertebrate Genomes
Source: G3 (Bethesda). 2015 Jan 15;5(3):441–7. doi: 10.1534/g3.114.015545 (PMC4349097; doi:10.1534/g3.114.015545)
Supplement: Supporting Information [file supp_5_3_441__index.html]

Evolutionary Consequences of DNA Methylation on the GC Content in Vertebrate Genomes — Supporting Information 

# Evolutionary Consequences of DNA Methylation on the GC Content in Vertebrate Genomes

## Supporting Information for Mugal *et al.*, 2015

**Files in this Data Supplement:**

- Supporting Information - Tables S1-S2 (PDF, 87 KB)
- Table S1 - Multiple Linear Regression (MLR) analysis of CpG → CpA/TpG substitution rate in relation to CpG methylation level and female recombination rate. (PDF, 15 KB)
- Table S2 - Multiple Linear Regression (MLR) analysis of CpG → CpA/TpG substitution rate in relation to CpG methylation level and male recombination rate. (PDF, 21 KB)
