# Supplementary material for: Evolutionary Consequences of DNA Methylation on the GC Content in Vertebrate Genomes
Source: G3 (Bethesda). 2015 Jan 15;5(3):441–7. doi: 10.1534/g3.114.015545 (PMC4349097; doi:10.1534/g3.114.015545)
Supplement: Supporting Information [file supp_g3.114.015545_015545SI.pdf]

G3 · Genes | Genomes | Genetics

Evolutionary consequences of DNA methylation on the GC content in vertebrate genomes

Carina F. Mugal[*], Peter F. Arndt[§], Lena Holm[†] and Hans Ellegren[*]

[*] Dept of Ecology and Genetics, Uppsala University, Norbyvägen 18D, SE-752 36 Uppsala, Sweden

[§] Department of Computational Molecular Biology, Max Planck Institute for Molecular Genetics, Ihnestrasse 63/73, DE-14195 Berlin, Germany

[†] Dept of Anatomy, Physiology and Biochemistry, Swedish University of Agricultural Sciences, Ulls väg 27 D-F, SE-756 51 Uppsala, Sweden

Data accession

Chicken bisulfite sequencing data have been deposited in GEO with accession GSE56639.

Author of correspondence

Carina F. Mugal, Dept of Ecology and Genetics, Uppsala University, Norbyvägen 18D, SE-752 36 Uppsala, Sweden, Phone: +46-18-4716417, Fax: +46-18-4716310, Email: carina.mugal@ebc.uu.se

DOI: 10.1534/g3.114.015545

Table S1: Multiple Linear Regression (MLR) analysis of CpG → CpA/TpG substitution rate in relation to CpG methylation level and female recombination rate. Partial correlations significant below a p-value threshold of 0.05 are printed in bold.

	chicken		human	
	partial correlation	*p*-value	partial correlation	*p*-value
CpG methylation level	**0.269**	$1.30 \cdot 10^{-15}$	**0.369**	$< 2 \cdot 10^{-16}$
Female recombination rate	**-0.436**	$< 2 \cdot 10^{-16}$	-0.029	$3.49 \cdot 10^{-1}$
	$R^2 = 0.27$		$R^2 = 0.14$	

Table S2: Multiple Linear Regression (MLR) analysis of CpG → CpA/TpG substitution rate in relation to CpG methylation level and male recombination rate. Partial correlations significant below a *p*-value threshold of 0.05 are printed in bold.

	chicken		human	
	partial correlation	*p*-value	partial correlation	*p*-value
CpG methylation level	**0.299**	$< 2 \cdot 10^{-16}$	**0.371**	$< 2 \cdot 10^{-16}$
Male recombination rate	**-0.490**	$< 2 \cdot 10^{-16}$	-0.057	$6.30 \cdot 10^{-2}$
	$R^2 = 0.32$		$R^2 = 0.14$	
