# Supplementary material for: Evolutionary Consequences of DNA Methylation on the GC Content in Vertebrate Genomes
Source: G3 (Bethesda). 2015 Jan 15;5(3):441–7. doi: 10.1534/g3.114.015545 (PMC4349097; doi:10.1534/g3.114.015545)
Supplement: Supporting Information [file supp_g3.114.015545_TableS1.pdf]

Table S1: Multiple Linear Regression (MLR) analysis of CpG → CpA/TpG substitution rate in relation to CpG methylation level and female recombination rate. Partial correlations significant below a p-value threshold of 0.05 are printed in bold.

	chicken		human	
	partial correlation	*p*-value	partial correlation	*p*-value
CpG methylation level	**0.269**	$1.30 \cdot 10^{-15}$	**0.369**	$< 2 \cdot 10^{-16}$
Female recombination rate	**-0.436**	$< 2 \cdot 10^{-16}$	-0.029	$3.49 \cdot 10^{-1}$
	$R^2 = 0.27$		$R^2 = 0.14$	
