# Supplementary material for: Evolutionary Consequences of DNA Methylation on the GC Content in Vertebrate Genomes
Source: G3 (Bethesda). 2015 Jan 15;5(3):441–7. doi: 10.1534/g3.114.015545 (PMC4349097; doi:10.1534/g3.114.015545)
Supplement: Supporting Information [file supp_g3.114.015545_TableS2.pdf]

Table S2: Multiple Linear Regression (MLR) analysis of CpG → CpA/TpG substitution rate in relation to CpG methylation level and male recombination rate. Partial correlations significant below a *p*-value threshold of 0.05 are printed in bold.

| | chicken | | human | |
|---|---|---|---|---|
| | partial correlation | *p*-value | partial correlation | *p*-value |
| CpG methylation level | **0.299** | $< 2\cdot10^{-16}$ | **0.371** | $< 2\cdot10^{-16}$ |
| Male recombination rate | **-0.490** | $< 2\cdot10^{-16}$ | -0.057 | $6.30\cdot10^{-2}$ |
| | $R^2 = 0.32$ | | $R^2 = 0.14$ | |
